# Detection of Novel Potential Regulators of Stem Cell Differentiation and Cardiogenesis through Combined Genome-Wide Profiling of Protein-Coding Transcripts and microRNAs

**DOI:** 10.3390/cells10092477

**Published:** 2021-09-18

**Authors:** Rui Machado, Agapios Sachinidis, Matthias E. Futschik

**Affiliations:** 1 Systems Biology and Bioinformatics Laboratory (SysBioLab), Center for Biomedical Research (CBMR), University of Algarve, Campus de Gambelas, 8005-139 Faro, Portugal; rsmachado@ualg.pt; 2 Working Group Sachinidis, Center for Physiology, University of Cologne, Faculty of Medicine and University Hospital Cologne, 50931 Cologne, Germany; 3 Center for Molecular Medicine Cologne, University of Cologne, 50931 Cologne, Germany; 4 Centre of Marine Sciences (CCMAR), University of Algarve, 8005-139 Faro, Portugal; 5 MRC London Institute of Medical Sciences (LMS), Faculty of Medicine, Imperial College London, Hammersmith Hospital Campus, Du Cane Road, London W12 0NN, UK

**Keywords:** stem cell differentiation, gene regulation, microRNAs, transcriptomics, cardiogenesis

## Abstract

In vitro differentiation of embryonic stem cells (ESCs) provides a convenient basis for the study of microRNA-based gene regulation that is relevant for early cardiogenic processes. However, to which degree insights gained from in vitro differentiation models can be readily transferred to the in vivo system remains unclear. In this study, we profiled simultaneous genome-wide measurements of mRNAs and microRNAs (miRNAs) of differentiating murine ESCs (mESCs) and integrated putative miRNA-gene interactions to assess miRNA-driven gene regulation. To identify interactions conserved between in vivo and in vitro, we combined our analysis with a recent transcriptomic study of early murine heart development in vivo. We detected over 200 putative miRNA–mRNA interactions with conserved expression patterns that were indicative of gene regulation across the in vitro and in vivo studies. A substantial proportion of candidate interactions have been already linked to cardiogenesis, supporting the validity of our approach. Notably, we also detected miRNAs with expression patterns that closely resembled those of key developmental transcription factors. The approach taken in this study enabled the identification of miRNA interactions in in vitro models with potential relevance for early cardiogenic development. Such comparative approaches will be important for the faithful application of stem cells in cardiovascular research.

## 1. Introduction

Embryonic stem cells (ESCs) are derived from the inner cell mass and can differentiate into derivatives of endodermal, ectodermal and mesodermal cell lineages, which ultimately leads to the generation of specialised somatic cell types [1]. Using the hanging drop technique, it is possible to simulate early embryonic development processes, as cells aggregate at the tip of the droplet through gravitational force, inducing the cells to differentiate and to form embryoid bodies (EBs) [1]. The EBs contain non-patterned and organised cell clusters that retain organoid characteristics, such as multiple specific cell types, with the capability of recapitulating some specific organ functions [2]. EBs are particularly important models for the study of cardiomyogenesis, due to the early emergence of foci composed by beating cardiomyocytes [3].

During the differentiation process, the expression of numerous genes needs to be regulated to tightly control and orchestrate various biological processes including intercellular communication, cell–cell interactions and apoptosis [4,5]. To understand the underlying gene regulation, functional genomic technologies, such as high throughput assays and microarrays, have helped to identify key transcription regulators, such as transcription factors, non-coding mRNAs and microRNAs (miRNAs) [6,7]. While transcription factors (TFs) guide the initiation of transcription, miRNAs commonly regulate gene expression at the post-transcriptional level by targeting mRNAs through imperfect base pairing [8], which leads to translational inhibition and/or mRNA degradation [9]. Formation of mature miRNAs occurs in four steps: (1) formation of a primary miRNA (pri-miRNA) via RNA polymerase II; (2) processing of the pri-miRNA by Drosha (an RNase III type endonuclease) and the double-stranded RNA binding protein DGCR8 (DiGeorge syndrome critical region gene 8) complex to produce a hairpin precursor miRNA (pre-miRNA); (3) export of the pre-miRNA to the cytosol via the protein exportin-5; (4) cleavage by Dicer (an RNase III type endonuclease) to produce ~22-bp double-stranded miRNA [10]. Following this final step, one strand is typically degraded, while the other strand is integrated into the RNA-induced silencing complex (RISC). The RISC targets complementary sequences in the mRNAs that are mainly located in the 3′ untranslated region (3′-UTR) but can also be found in the 5′-UTR and coding regions, resulting in mRNA degradation or inhibition of its translation [10].

The importance of a number of miRNAs for the differentiation of various cell types, including ESCs, has been well established [11,12,13,14,15]. To gain deeper insights in the dynamics of miRNAs and their impact on potential target genes, transcriptomics studies with parallel measurements of miRNAs and mRNAs have been particularly useful [16]. Hence, we applied genome-wide profiling of miRNAs and mRNAs that were simultaneously measured during in vitro differentiation of murine ESCs over a period of 16 days. Profiles of mRNA and miRNA were subsequently analysed and their differential expression was determined. Remarkably, we could identify miRNAs displaying characteristic peaks that resemble the transient expression patterns of key developmental TFss and could indicate a potential role as molecular switches. Due to the relevance of the EB model for the study of cardiogenesis and cardiovascular research, we aimed to unveil novel miRNAs that show regulatory patterns conserved between in vitro differentiation and in vivo heart development [16,17,18]. To this end, we compared the in vitro results with a recent transcriptomics study of murine fetal heart development, which captured both miRNA and mRNA levels using the same microarray platforms [16]. This led to the discovery of over 200 putative miRNA-gene interactions with expression patterns that indicate a conserved regulation between in vivo and in vitro systems. We suggest that these interactions are formidable candidates for further functional validation studies in vitro with direct relevance for cardiac gene regulation in vivo.

## 2. Materials and Methods

### 2.1. Culturing and Differentiation Assays of ESCs

Culturing and differentiation of murine CGR8 ESC from the European Collection of Cell Cultures (ECACC; No. 95011018) were performed as described previously [19]. Briefly, cells were maintained on (0.2%) gelatinized tissue-culture dishes under feeder-free conditions in standard ESC culture medium, which consisted of Glasgow’s minimum essential medium (GMEM; Invitrogen, Waltham, MA, USA) supplemented with 10% fetal bovine serum (FBS; GIBCO, Thermo Fisher Scientific, Waltham, MA, USA), 2 mM L-glutamine, 100 units/mL leukemia inhibitory factor (LIF-1; Calbiochem, San Diego, CA, USA) and 50 μM β-mercaptoethanol (β-ME; Invitrogen). Passaging of cells was carried out on alternate days with their confluence maintained between 60–70%. ESC differentiation was inducted by the previously described hanging drop protocol [20]. To this end, 20-μL hanging drops were generated in 10-cm diameter low adhesion dishes from a trypsin-dissociated ESC suspension (2.5 × 104 cells/mL), prepared in differentiation medium (Iscove’s modified Dulbecco’s Medium, IMDM; Life Technologies, Carlsbad, CA, USA), supplemented with 20% fetal calf serum, 1% non-essential amino acids, 2 mM L-glutamine and 100 μM β-ME. Plates were incubated at 37 °C and under 5% CO_2_ in a humidified incubator for 2 days. EBs formed were harvested through washing and resuspended in differentiation medium. The EBs were subsequently incubated at 37 °C under 5% CO_2_ in an incubator under shaking conditions, with a medium change on alternate days.

### 2.2. Microarray Data Processing and Data Quality Assessment

For mRNA and miRNA profiling, Affymetrix Mouse Genome 430 2.0 and Affymetrix miRNA 3.0 arrays were used, respectively. Microarray data were processed using the robust multi-array average (RMA) method implemented in the affy R/Bioconductor package. To reduce noise in the data, an expression threshold was set for genes and miRNAs. Genes and miRNAs were considered to be expressed and included for further analysis if the log2 intensity produced by the RMA for the corresponding probes was larger than five in at least one time point. Affymetrix Mouse Genome 430 2.0 arrays contained a total of 45,101 probe sets covering 20,702 genes with unique Entrez Gene IDs, of which 13,588 were found to be expressed. Likewise, Affymetrix miRNA 3.0 arrays contained a total of 1966 probe sets targeting miRNAs associated to Mus musculus, of which 415 were expressed. Sample variability was assessed using principal component analysis (PCA,) hierarchical clustering or density plots, implemented in R. Differential expression analysis was performed using the Bioconductor package limma [21]. Samples from day 0 (ESCs) were used as reference. Genes and miRNAs were defined as differentially expressed, when the corresponding adjusted *p*-value was lower than 10^−5^ and an absolute log2 fold change of expression of greater than 2 was recorded. Microarray measurements from the time series experiments were previously confirmed by RT-qPCR for selected marker genes and miRNAs [19].

### 2.3. Determining miRNA Peak Patterns

To identify well-defined miRNA expression peak for each day, we calculated the cross-correlation of the expression with binary vectors for the different time points. For instance, to find peaks at day 0, expression values were correlated with the vector (1,0,0,0,0). To find peak expression at day 4, the binary vector was set to (0,1,0,0,0) and so forth. To measure for correlation, the Pearson correlation coefficient was used. Thus, a miRNA achieved a large positive correlation if it was highly expressed at a single time point, while expressed at baseline level for the remaining time points.

### 2.4. Functional Enrichment Analysis

Gene ontology (GO) enrichment analysis was carried out in R applying an in-house script that uses the Bioconductor packages org.Mm.eg.db [22] and GOstats [23]. For testing of overrepresentation (enrichment) of genes associated with a GO category, the hypergeometric test, implemented as hyperGtest function in GOstats, was applied [23]. One challenge in the use of GO for enrichment analysis is the detection of highly overlapping categories due to the hierarchical structure of GO. To reduce the number of similar categories detected as enriched, a hypergeometric test conditioned to the GO tree structure was applied [23]. More specifically, the content of significant child terms (*p*-value ≤ 0.01) was excluded from testing. In this way, the test assesses for each category whether there exists evidence for enrichment beyond that provided by more specific child categories.

### 2.5. Integrative Analysis of Expression Data and Potential Regulatory Interactions

To obtain a comprehensive set of putative miRNA–mRNA interactions, data from five resources were merged: microRNA.org [24], Pita [25], miRDB [26], TargetScan [27] and MirTarBase [28]. The first four resources provided computationally predicted interactions while the latter included interactions based on experimental evidence. To obtain interactions of high confidence, the computationally predicted interactions were filtered following the recommendations of the creators of the resources (as previously described in Sabour et al., 2018 [16]). Interactions were discarded if they contained miRNAs and mRNAs that were not expressed. Integration and filtering resulted in a list of 104,001 potential interactions between 319 miRNAs and 10,594 target genes. Correlation between mRNAs and miRNAs was calculated using Kendall rank correlation, which provides a robust measure of the similarity of expression.

## 3. Results

### 3.1. Differential Gene Expression during In Vitro Differentiation of ESCs

**Figure 1:**
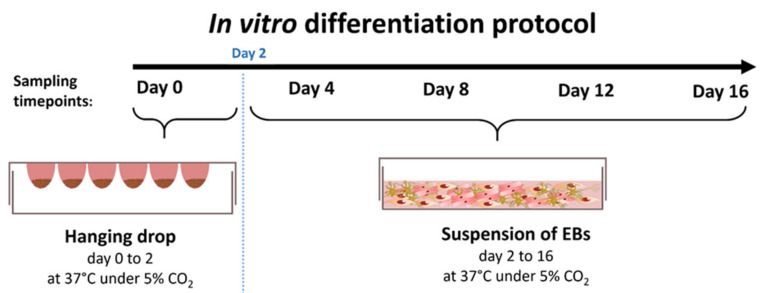
Time series experiment for simultaneously profiling mRNAs and microRNAs (miRNAs) during in vitro stem cell differentiation. Embryonic body (EB) formation was inducted over two days using the hanging drop approach. Samples for transcriptomic profiling were taken at days 0, 4, 8, 12 and 16. For images of the morphological changes of EB during stem cell differentiation, the interested reader is referred to a previous publication [4].

The basis of our study were time series experiments for ESCs with cells collected at days 0, 4, 8, 12 and 16 (Figure 1). Three replicate experiments were conducted resulting in a total of 15 samples. The mRNA and miRNA content of each sample was profiled using Affymetrix Mouse Genome 430 2.0 Arrays and Affymetrix miRNA 3.0 GeneChips.

**Figure 2:**
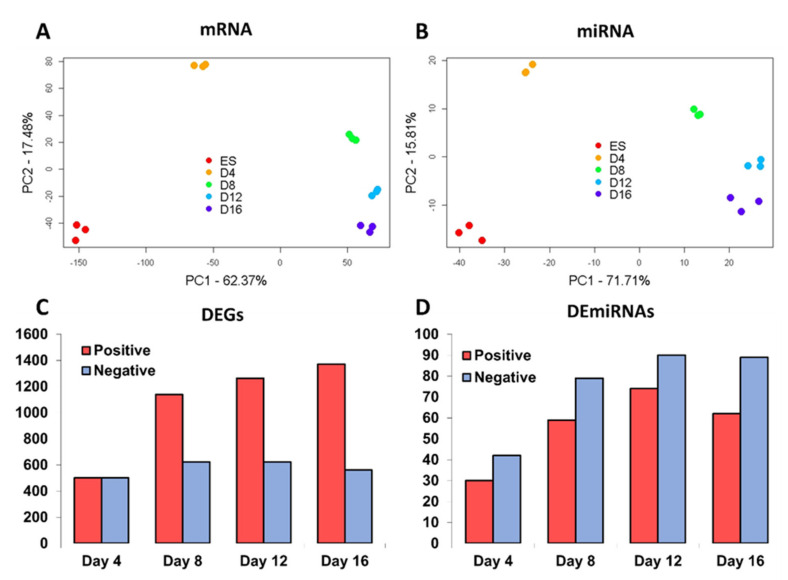
mRNA and miRNA profile analysis of in vitro ESCs differentiation. (**A**,**B**) Principal component analysis (PCA) of mRNA and miRNAs profiles. The two principal components (PC) capturing the largest variance for mRNA and miRNA are displayed. (**C**,**D**) Number of differentially expressed genes (DEGs) and miRNAs (DEmiRs) with positive or negative log2 fold changes in expression with day 0 as reference.

To gain a first overview of the transcriptomic changes measured in the experiment, we carried out a PCA analysis. Samples taken at the same time point were in proximity to each other, while samples from different time points were placed apart, indicating the reproducibility of measurement and the presence of a robust temporal expression signature (Figure 2A,B).

The largest differences between time points were observed for the first half of the time series, i.e., between day zero and day eight. After day eight, the variation diminished and the transcriptome profiles for mRNAs and miRNAs tended to stabilise. To identify differentially expressed genes (DEGs), normalised Affymetrix GeneChip signal intensities measured at days 4, 8, 12 and 16 were compared to the intensities of day zero.

A threshold for differential expression was set with an adjusted p-value of ≤10^−5^ and an absolute log2 fold change of ≥2 (4-fold change). The stringent threshold was motivated by the large number of genes that displayed changes in expression. In total, 2718 non-redundant genes were detected as differentially expressed for at least one time point, representing a total of 13% of all genes covered by the array. The number of DEGs doubled from 1003 at day four to 1933 at day 16 (Figure 2C). Notably, we found that considerably more genes were upregulated than downregulated at days 8, 12 and 16 compared to day zero. For day four, no such tendency was observed. A full list of DEGs at each time point is provided in Table S1.

For detection of differentially expressed miRNAs (DEmiRs), we used the same thresholds and reference time point as for the identification of DEGs. This led to the detection of 196 DEmiRs in total. Similar to DEGs, the number of DEmiRs increased during ongoing differentiation from 72 DEmiRs on day four to 151 DEmiRs on day 16 (Figure 2D). Contrary to DEGs, more negatively than positively regulated miRNAs were detected. The list of DEmiRs can be found in Table S2.

### 3.2. Dynamic Expression of Marker Genes and miRNAs

**Figure 3:**
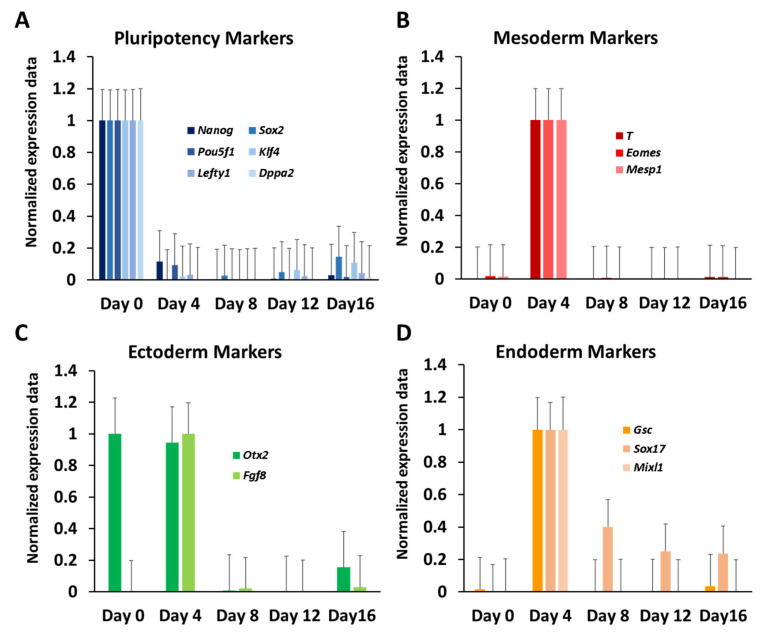
Expression of marker genes. (**A**) Pluripotency markers; (**B**–**D**) mesoderm, ectoderm and endoderm markers. Expression values for each gene were normalised to have a minimum value of 0 and a maximum value of 1. Standard error bars are shown.

Differentiation of ESCs is characterised by the diminishing expression of master regulators of pluripotency and the sequential activation of TFs establishing specific germ layers and cell lineages [29]. To validate whether our time series experiment faithfully reproduced these features, we inspected the expression of known marker genes. To facilitate this analysis, transcript values were linearly transformed to a zero to one scale (Min–Max normalisation). As pluripotency makers, *Nanog*, *Sox2*, *Pou5f1* (*Oct4*), *Klf4*, *Dppa2* and *Lefty1* were chosen [29,30]. For these markers, maximum expression was observed at day zero followed by a rapid decline in expression (Figure 3A).

At day four, none of the pluripotency markers showed more than 10% of its expression level measured at day zero, demonstrating an efficient abolishing of the pluripotency state by the hanging drop method. Subsequently, we assessed the expression of TFs associated with the formation of the three germ layers: *Otx2* and *Fgf8* for ectoderm [31,32], T-Brachyury, *Eomes* and *Mesp1* for mesoderm [33,34] and *Gsc*, *Sox17* and *Mixl1* for endoderm [35]. Most of the selected TFs exhibited a prominent peak in expression at day four and much lower expression at the time points before and after, indicating early specification into germ lineages in our experiments (Figure 3B–D). Expression of selected markers can be found in Table S3.

**Figure 4:**
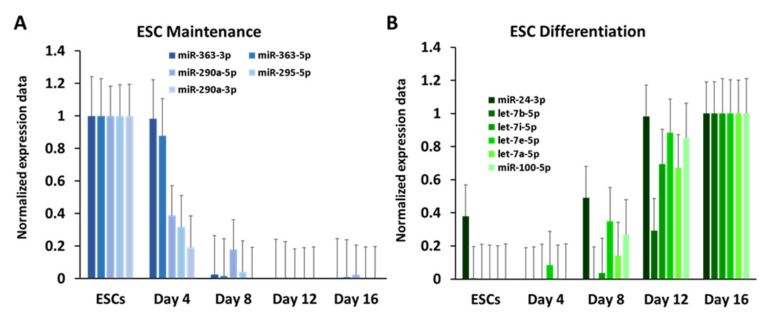
Expression of miRNAs markers. (**A**) Pluripotency miRNAs; (**B**) developmental miRNAs. Expression values for each gene were normalised to have a minimum value of 0 and a maximum value of 1. Standard error bars are shown.

To assess the data quality of miRNA measurements, we inspected expression values of miRNAs that have previously been linked to stem cell maintenance and differentiation. In particular, we examined the expression patterns of miR-290a-3p, miR-290a-5p, miR-295-5p, miR-363-3p and miR-363-5p, as they have been associated with stem cell maintenance and proliferation [13,14,15]. Similar to the verified pluripotency TFs, our data showed maximum expression at day zero and drastically lower expression at following time points (Figure 4A).

For assessment of miRNAs expected to be upregulated during differentiation we selected miR-24-3p, which has been reported to impinge on stem cell differentiation [36], let-7 family members, which are involved in embryogenesis [37], brain development [38] and hematopoietic stem cell fate [39] and miR-100-5p, which is required for ESC differentiation [40]. All of these miRNAs displayed a gradual increase in expression along the timeline and reached maximum abundance at day 16 (Figure 4B). Thus, the inspection of known markers indicated that both mRNA and miRNA levels measured in our microarray experiment agreed well with the findings of previous expression studies.

### 3.3. Elucidation of a Cardiogenic Signature in Embryonic Bodies

EBs generated through hanging drop technique are a well-established model to study differentiation of ESCs into more specialised cells. In particular, the formation of beating foci presents an easily trackable system for the study of cardiogenesis [41]. To elucidate this feature on a molecular level, we examined the expression of genes that are known to participate during heart morphogenesis, development, maturation and/or maintenance in our time series experiment.

**Figure 5:**
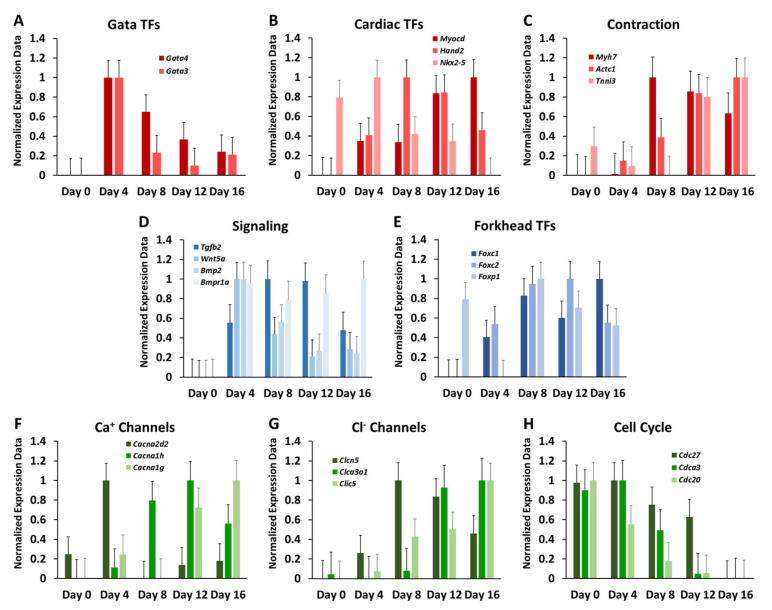
Temporal expression profiles of genes associated with heart development and ion channel function. Expression values were transformed to the [0, 1] range. The scale included a larger range to capture the errors intervals. In the graphics are shown: (**A**) GATA transcription factors (TFs); (**B**) other known key TFs for cardiogenesis; (**C**) genes encoding for structural cardiac proteins; (**D**) signalling pathway molecules linked for cardiac development; (**E**) forkhead gene family; (**F**) calcium channel genes; (**G**) chloride channel genes; (**H**) cell cycle genes. Standard error bars are shown.

To facilitate this analysis, transcript values were linearly transformed into a zero to one scale (Min–Max normalisation). First, we inspected the expression of cardiac TFs: *Gata3*, *Gata4* and *Nkx2-5* peaked at day four of stem cell differentiation, marking early cardiac progenitor cells in a precocious development state (Figure 5A,B), while *Hand2* and *Myocd* (transcriptional co-activator of the serum response factor) displayed an increase of expression at later time points (Figure 5B).

The latter indicates the presence of smooth and cardiac muscle cells in the differentiating stem cell population [42]. Subsequently, we examined the expression of genes associated with cardiac muscle contraction, which is the primary function of the heart (Figure 5C). *Myh7*, which encodes for the β-myosin heavy chain, was most strongly expressed at day eight and was gradually decreasing at later days, while *Actc1* and *Tnni3* displayed expression peaks at later stages of differentiation. These findings are consistent with previous observations of postnatal downregulation of *Myh7* in mice and other rodent hearts [43]. Strikingly, *Actc1*, a known marker for early myogenesis, had the highest signal intensity at later time points. Likewise, the expression of cardiac troponin I (*Tnni3*) was gradually upregulated during differentiation, with maximum expression occurring at day 16.

Members of BMP, Wnt and Tgf-β signalling pathways were also observed to be expressed during in vitro stem cell differentiation. These pathways have well established roles in heart development. *Tgf-β* is an important regulator of a wide range of cellular responses during cell differentiation [44], while BMP and WNT signalling pathways contribute, for instance, to specification of the first and second heart field progenitors [45]. All of the inspected signalling genes (*Tgf-β2*, *Wnt5a*, *Bmp2* and *Bmpr1a*) were not expressed in undifferentiated cells but displayed characteristic expression patterns at later time points. Both *Wnt5a* and *Bmp2* reached maximum expression at day four and were downregulated over time afterwards. *Tgf-β2* displayed an increased expression from day four until day twelve and decreased expression afterwards, while *Bmpr1a* showed stable expression levels from day four on (Figure 5D).

Expression of *Foxc1* and *Foxc2* of the forkhead family of TFs, which are known to be important factors in embryonic heart development [46], peaked at day 16 and day 12, respectively. *Foxc1* and *Foxc2* have non-redundant functions, since the elimination of both genes presents a more severe phenotype [47]. Furthermore, they contribute to the formation of the heart outflow tract and the right ventricle [48]. In contrast to *Foxc1* and *Foxc2*, *Foxp1* was expressed at day zero but not expressed at day four. Its expression exhibited a peak at day eight and was decreased progressively afterwards (Figure 5E). *Foxp1* is known to be downregulated during early differentiation [49] while still being essential at later stages for heart development, particularly in the myocardial area, valve formation and ventricular septation [50].

Components of ion channels also showed dynamic expression patterns. *Cacna2d2*, which encodes for α2-δ subunits of voltage-gated calcium (Ca^2+^) channels, peaked at day four and its expression was gradually decreased during differentiation, while Cacna1h encoding for α1 subunits peaked at day 12 and then decreased in expression (Figure 5F). In contrast, the gene *Cacna1g*, which is a paralog of *Cacna1h* and encodes an alternative α1 subunit, displayed a small increase in expression at day four and reached a maximum at day 18 only. Similarly, the chloride channels showed distinct dynamics, with *Clcn5*, *Clca3a1* and *Clic5* displaying the strongest increase in expression at day 4, day 8 and day 12, respectively (Figure 5G). *Clic5* is known to be expressed in cardiomyocyte mitochondria. Therefore, it might be expected to observe a peak in expression at later differentiation stages [51]. Finally, transcript levels of cell-division cycle associated genes (Figure 5H) decreased during stem cell differentiation with minimal expression at day 16. This reflects the loss of cell cycle activity with ongoing differentiation and might contribute to cell-cycle arrest of cardiomyocytes.

### 3.4. Identification of miRNAs That Might Serve as Genetic Switches

A striking feature of many key TFs for pluripotency maintenance or stem cell differentiation is well-defined transient activity, which is displayed as sharp expression peaks at a specific time point in our time series experiments (Figure 3). For instance, *Mesp1* showed a strong induction only at day four, while being expressed at low base level at all other time points (Figure 3B). Such binary, or ‘on–off’, expression patterns might reflect the role of these TFs as genetic switches during stem cell differentiation and embryonic development. This observation motivated us to examine whether such characteristic patterns also existed in the miRNA time series data, as they could point to miRNAs that might equally serve as genetic switches.

**Figure 6:**
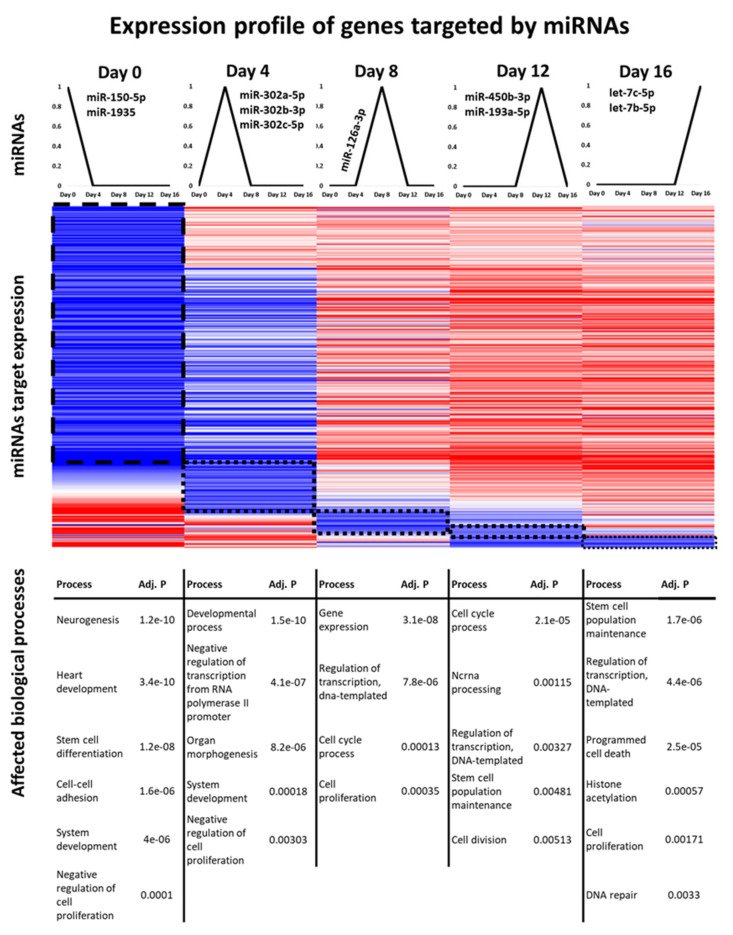
miRNAs target expression profiles. **Top panel**: Peak patterns used to identify miRNAs by cross-correlation analysis. Names for some identified miRNAs are shown. **Middle panel**: Heatmap for expression data of the miRNA targets. For visualisation, expression values were mean-centered. Shades of blue indicate expression values smaller than the gene-wise average of expression, while shades of red indicate expression values greater than the average expression. **Lower panel**: Results of Gene Ontology enrichment analysis of the top miRNAs targets.

To identify prominent peaks, the miRNA expression values were cross-correlated with binary vectors of the length of the time series (see Methods & Materials). Requiring a minimum correlation of 0.6 with the respective binary vector, cross-correlation identified 88 miRNAs with an expression peak at day 0, 16 miRNAs at day 4, 7 miRNAs at day 8, 4 miRNAs at day 12 and 13 miRNAs at day 16 (Figure 6, top panel). The identified miRNAs are listed in Table S4.

To gain further insight into the potential roles of the identified miRNAs, we analysed the functions of their targets based on high-confidence miRNA–mRNA interactions. To this end, we calculated the Kendall rank correlation coefficient τ for the gene expression levels of each miRNA–mRNA interaction and retained only miRNA–mRNA interactions with anti-correlated expression (τ < −0.2), as we assume effective regulation by miRNAs should be reflected by diminished mRNA levels of the target genes. For each time point, GO enrichment analysis was subsequently performed for target genes of the peaking miRNAs (Figure 6). To numerically balance the results, target genes of only the 10 miRNAs that showed the highest correlation with the binary vectors were included for days 0, 4 and 16.

This approach revealed a remarkable sequential order of repressed processes. Day zero exhibits the greatest number of negatively regulated targets, suggesting wide-ranging effects of the selected miRNAs through repressing genes that might cause stem cells to differentiate. Indeed, miRNAs with peak expression at day zero tended to silence processes related to morphogenesis and development such as neurogenesis or heart development. Furthermore, cell–cell adhesion and negative regulation of cell proliferation were preferentially targeted by the selected miRNAs. Most of these processes were still affected by miRNAs with peak expression at day four. The set of potentially repressed processes, however, changed drastically from day eight onwards. For the later time points, we observed a strong tendency of the selected miRNAs to target genes associated with cell cycle, cell proliferation, transcription and stem cell maintenance. Intriguingly, ncRNA processing is targeted by miRNAs peaking at day 12, suggesting potential negative feedback control on themselves. Finally, histone acetylation, which leads to opening of chromatin structures, appeared as significantly suppressed at day 16.

To further assess the potential importance of detected miRNAs, the number of target genes and targeted transcriptional regulators were derived from the miRNA–mRNA interaction data (Table S4). Notably, the number of targeted transcriptional regulators may give us an indication of the regulatory reach of a miRNA, as miRNAs can not only directly affect the abundance of transcriptional regulators themselves but also indirectly influence the abundance of the downstream targets of these regulators. Indeed, 47 of the 128 peaking miRNAs have more than 10 transcriptional regulators as potential targets. For instance, members of the let-7 family, which peaked at day 16, have each more than 15 transcriptional regulators as targets (mmu-let-7d-5p: 24 targeted transcriptional regulators, mmu-let-7b-5p: 22, mmu-let-7g-5p: 18, mmu-let-7c-5p: 17 and mmu-let-7f-5p: 15). This corresponds well with the established multifaceted roles of let-7 during differentiation, development and organ morphogenesis [52]. Intriguingly, a literature search for the miRNA peaking at days 4, 8 and 12 indicated that less than half of them have been associated already with stem cell differentiation and development (Table S4–Literature).

### 3.5. Detection of Shared In Vitro and In Vivo miRNA-mRNA Interactions

The detection of a prominent cardiogenic expression signature (Figure 5) supports the suitability of EBs for the study of cardiogenesis. In principle, therefore, EBs should provide means for the study of cardiac miRNAs and their targets genes through time series data analysis. Nevertheless, the heterogeneity of cell population in embryonic bodies imposes a challenge for interpretation and prioritisation of indicated interactions for further study, as anti-correlated expression of miRNAs and their targets could derive from regulatory activity in non-cardiac cells in EBs. To alleviate this problem, we used miRNA–mRNA interaction data derived previously for in vivo cardiogenesis in mice [16]. While heterogeneous cell populations in the in vitro experiment can affect gene/miRNA expression, we expect that gene/miRNA expression should be tightly associated with heart development and maturation for the in vivo experiment. The aim of the following analysis is to reveal which miRNA interactions are potentially active in vitro and in vivo during differentiation or development, respectively. Conserved interactions can then be prioritised for further study.

**Figure 7:**
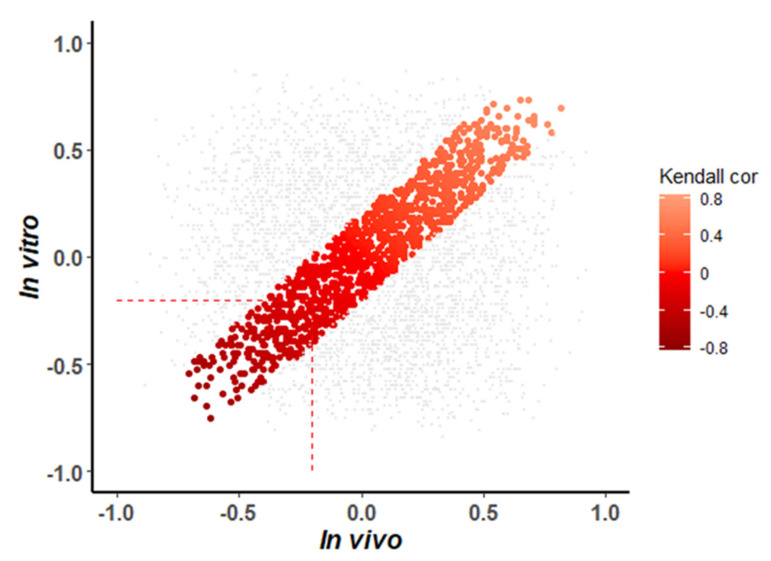
Correlation of miRNAs with their target genes for in vitro and in vivo experiments. Each dot represents a miRNA–mRNA interaction. The values of the *x*-axis are the Kendall correlation coefficients for the in vivo data; the values of the y-axis are the correlation coefficients for the in vitro data. Highlighted by red gradient colour are miRNA–mRNA interactions with similar correlation coefficients in both in vivo and in vitro, i.e., with a difference in correlation coefficient <0.2. Dashed lines indicate the threshold of negative correlation for prioritisation.

The analysis was based on the previously curated data set of miRNA–mRNA interactions [16], of potential interactions between 319 miRNAs and 10,596 target genes. We extracted all overlapping miRNA–mRNA interactions for both studies and obtained a table of 76,343 interactions. To identify relevant interactions, only interactions were kept for which miRNAs targeted genes associated with heart development in GO (GO:0007507) [53]. The additional filtering reduced the number of miRNA–mRNA interactions to 3983 interactions. For these interactions, correlation between miRNAs and target genes was calculated. The distribution of correlation coefficients for the in vitro and in vivo experiments is shown in Figure 7.

To identify potential interactions with a conserved correlation between miRNA and mRNA, we set a maximal threshold for the differences in correlation coefficients of 0.2. This led to the identification of 1129 interactions with conserved correlation. Assuming that the miRNAs tend to convey a repressive effect on target mRNAs, we selected miRNA–mRNA interactions with a negatively correlated expression (τ < −0.2) in both studies. This resulted in a set of 214 miRNA–mRNAs interacting pairs linking 102 unique miRNAs to 124 unique target genes (Table S5).

For further prioritisation, we first identified the miRNAs with the largest number of targets and verified whether they had been previously described in the literature to be associated with heart development (Table 1). From the top 20 miRNAs, 12 miRNAs (60%) have already been described as being associated with heart related events, either in development, maturation, differentiation or disease, supporting the chosen approach for prioritisation. These miRNAs included miR-27a-3p, mmu-miR-34a-5p and mmu-let-7b-3p. The remaining 40% of the miRNAs in Table 1 have not been yet associated with cardiac events in the literature. Examples are mmu-miR-680, mmu-miR-705 and mmu-miR-762.

Additionally, we compared the set of miRNAs involved in the 214 miRNA–mRNA interactions with the list of miRNAs prioritised in the in vivo study [16]. The prioritisation in the latter study was based on several criteria: (a) percentage of negatively regulated target genes among differentially expressed target genes as an estimate of regulatory activity; (b) number of negatively regulated transcriptional regulators as a measure for potential breadth of downstream effects; and (c) number of negatively regulated target genes associated with heart development. Comparison of the set of 102 miRNAs showed that almost two thirds (*n* = 66) were prioritised previously in the in vivo study, based on the different criteria (Table S6).

## 4. Discussion

How miRNAs contribute to stem cell differentiation towards cardiac fate is a challenging question. An answer could provide crucial clues not only for our understanding of heart development but for establishing faithful in vitro models for cardiac research. As in vitro models allow up-scaling, they are of particular importance when following up the numerous findings that typically derive from genomic in vivo studies. An example is presented by our previous parallel profiling of miRNA and mRNA levels in fetal murine hearts, where we found almost 200 miRNAs differentially expressed. One established in vitro model are EBs. They have been regularly used to study cardiomyogenic processes but are not without challenges, due to their cellular heterogeneity [3,41]. To dissect miRNA and gene expression data for EB formation and to gain relevant information for cardiac research, we applied an integrative approach combining expression analysis, interaction data, functional annotation and literature review. Various filtering and selection procedures were carried out to obtain candidate lists for future experimental characterisation. To minimise false positives, we applied stringent filters for expression and differential expression. As a trade-off, some prominent cardiac miRNAs, such as miR-1 or miR-133, were missing among DEmiRs due to the applied expression threshold (e.g., for miR-1) or threshold for differential expression (miR-133). A re-analysis of the publicly accessible microarray data with a more relaxed threshold could therefore offer the opportunity to enlarge the list of candidate miRNAs.

After verifying the successful induction of stem cell differentiation by inspection of the expression levels of marker genes and miRNAs, we explored the cardiac expression signature in EBs. Although caution needs to be taken in the interpretation of the observed expression values due to the confounding heterogeneric cellular composition of EBs, inspection of genes known to be associated with heart morphogenesis and function indicated the existence of a salient cardiac expression signature. Importantly, we observed the presence of TFs linked for heart development in the early time points, which indicates that a part of the cell population was differentiating towards a cardiac fate. The expressed TFs included GATA family members, *Hand2*, *Nkx2-5* and *Myocd*. We also identified the expression of genes associated with heart contraction and ion channels, pointing to the initiation of cardiac specification of cells. Collectively, the observations support the use of EBs for studying cardiogenesis, despite its challenges and restrictions [3,41]. Nevertheless, we also observed divergent expression patterns. For instance, *Actc1* showed its highest expression during early cardiogenesis and was repressed progressively during development in vivo [16], while higher levels of in vitro expression of *Actc1* were only detected at later stages of differentiation. This indicates that EB cells were, at least partially, still in an early maturation phase even at the final time point of the in vitro experiment.

Subsequently, we searched for miRNAs displaying characteristic peaks in expression, as they might serve as temporal switches during differentiation. Notably, several of the identified miRNAs have already been associated with development processes or used as biomarkers for the identification of different pathologies. Previous studies showed that the miR-302 family targets several genes that regulate cell cycle [54], and a decreased expression of members of this miRNA family affects early embryonic development [54]. In the context of heart development, miRNAs with peaks at days 8, 12 and 16 seem especially relevant. MiR-126-3p, peaking at day eight, was identified as a potential biomarker for coronary artery disease [55], while miR193a-5p with a peak at day 12 was shown to repress proliferation and migration of cardiac stem cells (CSCs) through the downregulation of *c-kit* [56]. Peaks at day 16 were detected for members of the let-7 family that have been linked to the regulation of multiple processes, including heart development and cardiac differentiation, as well as cardiovascular diseases [52]. For future studies, however, the candidates of greatest novelty might be constituted by miRNAs that have a large regulatory reach by targeting many transcriptional regulators but have not been assigned yet to a role in the context of stem cell differentiation. An example of such miRNA is miR-450b-3p, which peaks at day 12 and targets 18 transcriptional regulators, among which is *Klf4*, a TF for pluripotency. To our knowledge, a role for miR-450b-3p in the context of stem cell differentiation has not been described in literature. This suggests that the list of peaking, together with information about the targets (Table S4), offers a rich resource for follow-up studies.

We also examined the potential regulatory functions of these miRNA targets. The results were consistent with functional necessities of ESC differentiation. At day zero, processes associated with organogenesis and morphogenesis were inhibited, while affected processes could be linked to both maintenance and differentiation of the ESCs at day four. Processes related to cell cycle and pluripotency were effectively repressed after day eight.

Comparative analysis of in vitro and in vivo expression data enabled detecting similarities between studies and identification of miRNAs, which warrant further experimental validation. Such comparison was possible, as parallel profiling of miRNA and mRNA was carried out on the same microarray platform, minimising technical variability. To increase their relevance for cardiac research, we only included miRNA targets if they have been already associated with heart development in GO. This led to the detection of 1129 miRNA–mRNA interactions with conserved correlation of expression between studies. Remarkably, 214 miRNA–mRNA pairs showed a negative correlation of expression in both studies. Ranked by the number of target genes, we examined current knowledge for the top 20 miRNAs and found that 12 have been linked to heart development. Despite existing association, their role in heart development remains to be fully characterised, since many of the indicated miRNA–mRNA interactions await experimental validation (for more details see Table S6). One such miRNA is miR-680, which is predicted to target six genes involved in heart development. These targets include *Foxc1*, which is a known regulator of early cardiogenesis and controls the generation of functional cardiomyocytes together with other early cardiac TFs such as *Mef2c*, *Isl1* and *Nkx2-5* [57]. Furthermore, *Foxc1* regulates early cardiomyogenesis by actuating in a very specific time frame during differentiation, potentiating the efficiency of cardiomyocyte production from ESCs [57]. The targeting of *Tgfbr3* (transforming growth factor type III receptor, also known as betaglycan) by miR-680 can be a further interesting interaction for future validation, since TGFBR3 is an accessory co-receptor of TGF-β and is important for the maintenance and protection of cardiac fibroblasts [58]. While the regulatory role of TGFBR3 in the TGF-β signalling pathway is still not fully clear, recent results indicated TGFBR3 is a potential regulator of TGF-β signalling with anti-apoptotic properties in the heart [58]. In cardiac fibroblasts exposed to hypoxia, TGF-β signalling accounts predominantly for apoptosis of cardiac fibroblasts. However, the overexpression of *Tgfbr3* effectively prevented hypoxia-induced apoptosis of cardiac fibroblasts [58]. Other candidates for further study include miR-27a-3p and miR-23a-3p. The relevance of the former was already described in the literature [59,60], but the predicted interaction with *Mef2c* has not been experimentally validated yet. Similarly, the interaction of miR-23a-3p and *Isl1* awaits validation, with *Isl1* as a key transcription factor during early cardiac differentiation [61]. Finally, the list of the 20 top miRNAs includes eight miRNAs that have not yet been associated with cardiogenesis and thus appear as particularly attractive candidates for novel cardiac regulators.

Further support was gained through the comparison of the selected 102 miRNAs with conserved interactions to a list of miRNAs prioritised in the in vivo study (Table S6). For instance, miR-106a-5p, a member of the miR-17 family, has interactions and conserved negative correlation of expression with three target genes (*Heg1*, *Pkd1* and *Tgfbr2*) and was also prioritised in the in vivo study because of its potentially repressive interactions with heart developmental genes (*Heg1*, *Nrp2*, *Pkd1* and *Vegfa*) and TFs (*Ar*, *Hlf*, *Klf9*, *Stat3* and *Zbtb4*). Indeed, a relevance of miR-106a-5p for cardiogenesis was recently indicated experimentally, as overexpression of miR-106a-5p suppressed differentiation of C2C12 cells, a murine myoblast cell line [62]. There are also cases with strong converging support from both studies, but they are still lacking validation. An example warranting further study is mmu-miR-3472, for which we found three interactions of conserved negative correlation (*Sema3c*, *Tmem100* and *Foxc1*). Strikingly, miR-3472 has high regulatory activity predicted for the in vivo time series and targets both *Smad3* and *Foxc1* TFs. A critical role of *Foxc1* for heart development has been well established [57,63].

To gain biomedical relevance, identified processes and interactions need to be conserved in humans. While an exact determination of conservation is a challenging task and will require experimental efforts, we attempted to get a first insight into the degree of conservation based on available data for human miRNA–mRNA interactions and gene expression. We found 87 out of 214 miRNA–mRNA interactions conserved between in vivo and in vitro were also predicted to occur in humans (Table S5). This constitutes 40.5% of miRNA–mRNA interactions. Using experimentally supported human miRNA–mRNA interactions from miRTarBase, we found that 30 out of 214 miRNA–mRNA interactions are indicated to exist in humans. Next, we assessed whether genes targeted by miRNAs show conserved differential expression in human EBs during stem cell differentiation, based on the study by Gabdoulline and their co-workers [64]. In total, 62 out of 123 target genes (50.4%) were also detected as differentially expressed in human EBs. Importantly, the key cardiac transcription factors, such as *Mef2c*, *Foxc1* and *Gata3*, were found differentially expressed in both our murine EB as well as the human EB time series data.

Translation of our findings into the clinical context and the application of miRNA as a novel means in cardiac regenerative medicine is a crucial task but also a formidable challenge. We postulate that the detected miRNAs can help to control cardiac differentiation processes in vitro and in vivo, and thus could be powerful vehicles to improve the fidelity of in vitro models as well as to enhance regenerative treatments. Indeed, the application of miRNAs has already been expedited in the context of cardiac regenerative medicine for in vitro as well as in vivo models. The results demonstrated the possibility of inducing direct conversion of cardiac fibroblasts to cardiomyocytes by transient transfection with a small number of selected miRNAs after cardiac injury [65,66]. Additionally, miRNAs, such as the let-7 family members, can be used for enhanced maturation of cardiomyocyte-like cells in stem cell-derived cardiomyocytes cultures [67]. Our study augments considerably the number of miRNA candidates, which might prove to be useful for these objectives. For instance, the detection of let-7 among late peaking miRNAs agrees with a previous finding of enhanced cardiomyocyte maturation [67] and thus indicates that the other miRNAs peaking at day 16 can be relevant for the same purpose.

While the experiments indicated that these miRNA-based treatments can lead to improved cardiac function both for small and large mammals [66,68], caution is required, as a tightly controlled dosage of the miRNA transfection appears to be critical for long term success [68]. However, thanks to the immense recent interest and breath-taking progress made in constructing efficient delivery systems for in vivo expression of exogenous mRNA in humans as COVID-19 vaccines [69], we might expect that this acquired knowledge will nurture improvements in the delivery systems for miRNA-based therapies in the near future.

In summary, our parallel miRNA–mRNA profiling study provides novels insight into differentiation mechanisms of mESCs regulated by miRNAs. The study identified miRNAs that have transient expression patterns (peaks) similar to those of key developmental TFs and thus may equally act as important genetic switches. The study identified 200 putative miRNA–mRNA interactions with temporal expression patterns, which are of high relevance for in vivo developmental and differentiation processes. Moreover, it delivers a look-up atlas for cardiac miRNAs with conserved potential action in vitro and in vivo. Therefore, our study can serve as a Rosetta stone for the translation of miRNA-based regulation from a complex in vivo system into trackable in vitro models and vice versa. In conclusion, the present study can help to transfer knowledge from the in vitro to the in vivo situation.

## Figures and Tables

**Table 1:** Top 20 miRNAs targeting heart related genes both in vivo and in vitro. Some of the miRNA–mRNA interactions have been already experimentally tested but have not been associated with heart related processes. PMID column indicates the publication where an association has been already described.

miRID	Count	Gene Symbol	PMID
mmu-miR-27a-3p	7	*Robo1*,* Gata3*,* Zfp36l1*,* Flrt2*,* Mef2c*,* Slc8a1*,* Bmpr1a*	28293796
mmu-miR-34a-5p	7	*Pofut1*,* Fat4*,* Tab2*,* Maml1*,* Angpt1*,* Pdgfra*,* Kdm2a*	30474870
mmu-miR-680	6	*Ctnnb1*,* Zfp36l1*,* Foxc1*,* Gnaq*,* Zmiz1*,* Tgfbr3*	
mmu-let-7b-3p	6	*Sfrp2*,* Lmo4*,* Vegfa*,* Hectd1*,* Acvr1*,* Frs2*	27072074
mmu-miR-23b-3p	5	*Has2*,* Pcsk5*,* Adam19*,* Whsc1*,* Nox4*	31103821
mmu-miR-705	5	*Ncam1*,* Sh3pxd2b*,* Kdm2a*,* Casp7*,* Gpc3*	-
mmu-miR-23a-3p	5	*Isl1*,* Sox11*,* Robo1*,* Aldh1a2*,* Med12*	31130720
mmu-miR-762	5	*Ncam1*,* Pdpn*,* Tgfbr1*,* Zfp36l1*,* Nedd4*	
mmu-miR-27b-3p	4	*Smad4*,* Pdgfra*,* Ccm2*,* Tgfbr3*	27072074
mmu-miR-20b-5p	4	*Heg1*,* Pkd1*,* Nrp2*,* Tgfbr2*	25898012
mmu-miR-150-5p	4	*Efnb2*,* Prkar1a*,* Trps1*,* Erbb2*	31122130
mmu-miR-152-3p	4	*Dicer1*,* Ube4b*,* Gys1*,* Sos1*	-
mmu-miR-3473a	4	*Vegfa*,* Gab1*,* Camk2d*,* Mdm4*	-
mmu-miR-466f-3p	4	*Pitx2*,* Wisp1*,* Zfpm2*,* Kif3a*	-
mmu-miR-106b-5p	4	*Pkd1*,* Heg1*,* Tgfbr2*,* Grhl2*	-
mmu-miR-148a-3p	3	*Dicer1*,* Ube4b*,* Adam19*	25630970
mmu-miR-30c-5p	3	*Nfatc3*,* Adam19*,* Bcor*	30279543
mmu-miR-19b-3p	3	*Mef2a*,* Tgfbr2*,* Sgcb*	29664809
mmu-miR-3472	3	*Sema3c*,* Tmem100*,* Foxc1*	-
mmu-miR-106a-5p	3	*Heg1*,* Pkd1*,* Tgfbr2*	30004470

## Data Availability

The data presented in this study are openly available in the NCBI Gene Expression Omnibus under reference numbers GSE96808 and GSE96809.

## Supplementary Materials

The following are available online at www.mdpi.com/article/10.3390/cells10092477/s1, Table S1: List of differentially expressed genes for the day 4, 8, 12 and 16. Each spreadsheet includes the output of the Limma analysis: gene symbol, gene name, EntrezGene ID, log2 fold change (with respect to day 0), average log2 expression (of relevant time point and day 0), t statistics, p value and Benjamini-Hochberg adjusted p-value. Table S2: List of differentially expressed miRNAs for the day 4, 8, 12 and 16. Each spreadsheet includes the output of the Limma analysis: Affymetrix probeset ID, miRBase ID, log2 fold change (with respect to day 0), average log2 expression (of relevant time point and day 0), t statistics, p value and Benjamini-Hochberg adjusted *p*-value. Table S3: Stem cell and developmental marker genes and miRNAs displayed in Figure 3 and Figure 4. Both original and min-max normalized expression values are provided. Table S4: List of miRNAs with detected peak profiles for day 0, 4, 8, 12 and 16. The spreadsheets list the Affymetrix probeset IDs, the miRBase IDs, log2 expression, Kendall correlation values for binary vectors for different days, set and number of target genes and targeted transcriptional regulators. Table S5: Set of miRNA-mRNA interactions with conserved negative correlation in in vivo and in vitro time series experiments and that target genes associated with heart development (HD) in Gene Ontology. miRBase IDs, gene symbols and EntrezGene IDs, Kendall correlation coefficients for in vitro and in vivo timeseries are included. Association with HD or TF activity are indicated in the relevant columns. Additionally, PubMed IDs (PMIDs) are provided pointing to literature, which links the corresponding miRNA to cardiac development or phenotypes. Furthermore, indications are given whether the miR-miRNA interaction was found for human in the miRDB and miRTarBase, and whether the target gene was found differentially expressed in human EBs (Table S3 in [64]). Table S6: List of miRNAs with conserved negative correlation with target genes (TGs). It includes: miRbase IDs, count (number of TGs), gene symbols and EntrezGene IDs of TGs, literature references (PMID) for miRNAs. The list is integrated with data from in vitro study of murine heart development: number of TGs, number of differentially expressed TG (DETG), number of DETG with negative correlation to miRNA (DETGNC), numbers, gene symbols and EntrezGene IDs of DETGNCs that are associated with HD or TF activity.
